# Relationship between A1166C polymorphism of angiotensin II type 1 receptor gene and arteriosclerosis: A protocol for systematic review and meta-analysis

**DOI:** 10.1097/MD.0000000000024407

**Published:** 2021-01-29

**Authors:** Zhongping Shi, Jun Wang, Shanjiang Chen, Haiyue Dai, Yiwei Huang

**Affiliations:** Wenzhou Central Hospital, Wenzhou, Zhejiang Province, China.

**Keywords:** angiotensin II type1 receptor, arteriosclerosis, gene polymorphism, protocol, systematic review

## Abstract

**Background:**

Arteriosclerosis has genetic correlation. Many studies have shown that angiotensin II type 1 receptor (AT1R) gene A1166C polymorphism is highly associated with arteriosclerosis, but there is no evidence-based basis. The purpose of this study is to systematically evaluate the relationship between AT1R gene A1166C polymorphism and arteriosclerosis.

**Methods:**

The search time is set from the establishment of the database in December 2020 in this study. The search database include China National Knowledge Infrastructure (CNKI), Wanfang, VIP and China Biology Medicine disc (CBM), PubMed, EMBASE, Web of Science, and the Cochrane Library. The subjects are observational studies on the relationship between AGTR1 A1166C polymorphism and arteriosclerosis (including case-control study, cross-sectional study, and cohort study). The language is limited to English and Chinese. The data of the included study are extracted and the literature quality is evaluated by 2 researchers independently. The data are statistically analyzed by Stata 16.0 software.

**Results:**

This study will use pulse wave velocity as an index to evaluate arteriosclerosis to explore the relationship between AT1R gene A1166C polymorphism and arteriosclerosis.

**Conclusion:**

This study will provide evidence-based medicine for elucidating the genetic tendency of arteriosclerosis.

**Ethics and dissemination:**

Private information from individuals will not be published. This systematic review also does not involve endangering participant rights. Ethical approval will not be required. The results may be published in a peer-reviewed journal or disseminated at relevant conferences.

**OSF Registration number:**

DOI 10.17605/OSF.IO/V6E2Y

## 1 Introduction

Arteriosclerosis is an independent risk factor for cardiovascular events. It is the earliest clinical monitoring of vascular wall structure and functional changes.^[1]^ It is also a sign of the aging process, and the consequences of many disease states (such as diabetes, chronic renal insufficiency, atherosclerosis, etc).^[2]^ It is found that genetic factors play an important role in the occurrence and development of arteriosclerosis, which is not affected by blood pressure, heart rate, body weight, age, and other cardiovascular risk factors.^[3]^ Exploring the factors that affect the rhythm of arterial aging can help identify people who are at increased risk of cardiovascular disease.

Angiotensin II type 1 receptor (AT1R) is a G protein-coupled receptor, which mediates the physiological effects of vasomotor, water and salt metabolism, vascular smooth muscle cell proliferation and functional regulation, and is an important link in the role of angiotensin II.^[4]^ AT1R gene is located at 3q21-25, including 5 exons and 4 introns. AT1R A1166C polymorphism is located in the 3 ’non-transcribed region of the gene, which can interact with miR-155 and affect the expression of AT1R.^[5,6]^ Renin-angiotensin system plays an important role in regulating cell growth and differentiation, endothelial function, and cardiovascular homeostasis.^[7]^ Angiotensin receptor gene polymorphisms are widespread in the population. AT1R gene A1166C polymorphism (AGTR1 A1166C polymorphism) is due to the AT1R gene (located on the long arm of chromosome 3) 3’ the 1166th nucleotide in the noncoding region of the’ end was replaced by A-C.

Since the emergence of the first study in 1996 on the effects of angiotensin converting enzyme and AT1R gene polymorphism on normal blood pressure and aortic sclerosis in patients with hypertension,^[8]^ there have been a number of studies to explore the relationship between AGTR1 A1166C polymorphism and arteriosclerosis.^[2,9,10]^ However, due to differences in sample size, test efficiency, gene or environmental background, the results are not consistent. Therefore, we adopt the method of meta-analysis to comprehensively analyze the existing data, in order to provide evidence-based medical evidence for elucidating the genetic tendency of arteriosclerosis.

## 2 Methods

### 2.1 Protocol register

This protocol of systematic review and meta-analysis has been drafted under the guidance of the preferred reporting items for systematic reviews and meta-analyses protocols (PRISMA-P).^[11]^ Moreover, it has been registered on open science framework (OSF) on December 24, 2020 (Registration number: DOI 10.17605/OSF.IO/V6E2Y).

### 2.2 Ethics

Since the program does not require the recruitment of patients and the collection of personal information, it does not require the approval of the Ethics Committee.

### 2.3 Eligibility criteria

(1) The study evaluates the relationship between the AGTR1 A1166C polymorphism and arteriosclerosis;
(2) The study is an observational study (including case-control studies, cross-sectional studies, and cohort studies);
(3) The evaluation index of arteriosclerosis in the study is pulse wave velocity (PWV);
(4) Allele or genotype distribution frequency data is available;
(5) The distribution frequency of genotype conforms to Hardy-Weinberg's law.

### 2.4 Exclusion criteria

(1) Repeated published research;
(2) Articles in which the published literature is an abstract or review, the data of the article are incomplete or incorrect, and the complete data cannot be obtained after contacting the author;
(3) The study on the failure to provide detailed data on the frequency of genotypes;
(4) Literature without relevant outcome indicators;
(5) The objects of studying are not from human beings.

### 2.5 Retrieval strategy

Use “Angiotensin II type 1 receptor,” “Gene Polymorphism,” and “Arteriosclerosis” as Chinese search words in Chinese database, including CNKI, Wanfang data knowledge Service platform, VIP Chinese Journal Service platform (VIP), and China Biology Medicine disc (CBM). The English words “Angiotensin II type 1 receptor,” “AGTR1,” “Polymorphism,” “variant,” “arterial stiffness,” and so on will be searched in the English database, including PubMed, EMBASE, Web of Science, and the Cochrane Library. All the literature about the relationship between AGTR1 A1166C polymorphism and arteriosclerosis will be collected from the establishment of the database in December 2020. Take PubMed as an example, the retrieval strategy is shown in Table 1.

**Table 1:** Search strategy in PubMed database.

Number	Search terms
#1	angiotensin II type 1 receptor [Title/Abstract]
#2	AGTR1 [Title/Abstract]
#3	#1 OR #2
#4	polymorphism [Title/Abstract]
#5	variant [Title/Abstract]
#6	#4 OR #5
#7	Vascular Stiffness [MeSH]
#8	Arterial Stiffness [Title/Abstract]
#9	Aortic Stiffness [Title/Abstract]
#10	pulse wave velocity [Title/Abstract]
#11	PWV [Title/Abstract]
#12	#7 OR #8 OR #9 OR #10 OR #11
#13	#3 AND #6 AND #12

### 2.6 Data screening and extraction

**Figure 1:**
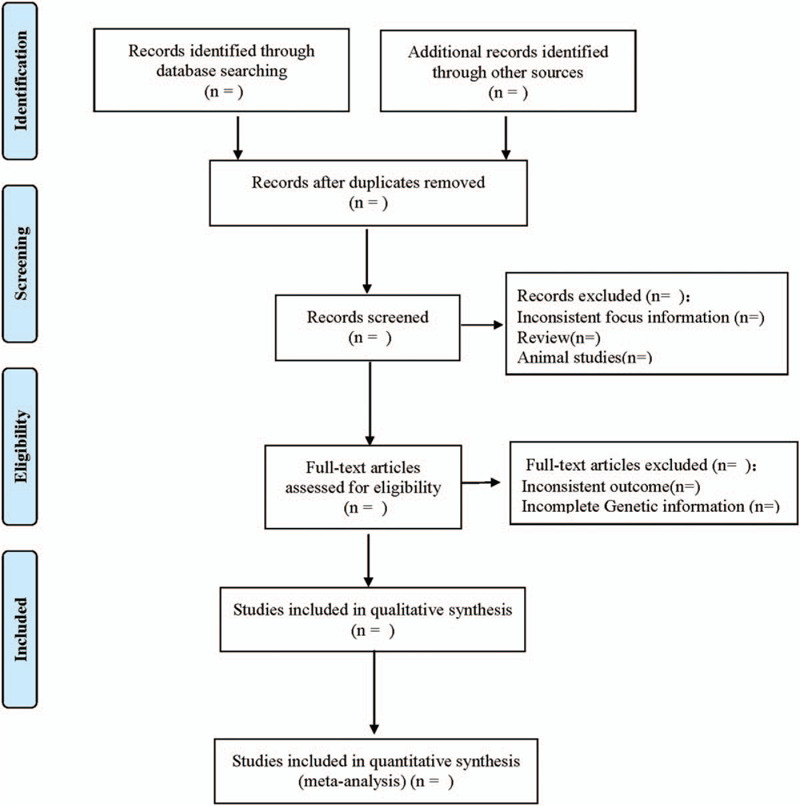
Flow diagram.

Two researchers independently complete the literature screening, exclude the studies that obviously do not meet the inclusion criteria, and further read the abstract and the full text to determine whether they meet the inclusion criteria. The data included in the literature will be extracted and cross-checked. In case of disagreement, consult with the third researcher and reach a consensus. The extracted data include: the first author, the number of years of publication, the country of publication, the race of the study population, the basic characteristics of the study population (including age, sex, disease, etc), the distribution of each phenotype of genes (whether obeying the law of Hardy- Weinberg equilibrium), the results of PWV and so on. The literature screening process is shown in Figure 1.

### 2.7 Literature quality assessment

The case-control study and cohort study will be evaluated by Newcastle-Ottawa Scale (NOS),^[12]^ including 3 columns and 8 items with a total of 9 points, and the evaluation criteria is ≥6 as high quality, while the cross-sectional study is evaluated with 11 standard items recommended by the Agency for Healthcare Research and Quality (AHRQ) to evaluate the cross-sectional study. Zero to three are classified as low quality, 4 to 7 as medium quality, 8 to 11 as high quality.^[13]^

### 2.8 Statistical analysis

#### 2.8.1 Data analysis and processing

Before evaluating the correlation between gene polymorphism and arteriosclerosis, χ^2^ will be used to test whether the genotype distribution in the control group is in accordance with Hardy-Weinberg genetic equilibrium (*P ≥* . 05). Meta-analysis is carried out by using STATA.16 software, continuous variables will be expressed in the form of standard mean deviation and 95% confidence interval, the binary classification variables will be expressed as odds ratio and 95% confidence interval. Heterogeneity is analyzed by *Q* test and *I*^2^, and the heterogeneity is evaluated according to the *I*^2^ value. If *P* *>* .1*, I*^*2*^ *<* 50%, the heterogeneity among the included studies is small, so the fixed effect model is used for analysis; if *P* *<* .1 and *I*^*2*^*≥* 50%, the heterogeneity between the included studies is obvious, and the sources of heterogeneity are analyzed. Random effect model will be used for analysis.

#### 2.8.2 Dealing with missing data

If the data of the required study are incomplete or not reported in the study, the researcher will contact the first author or other authors of the study by phone or email. If the required data are not available, we will use descriptive analysis instead of meta-analysis and exclude these studies if necessary.

#### 2.8.3 Subgroup analysis

In order to deal with the heterogeneity between different studies, subgroup analyses will be conducted according to ethnicity (such as Asians, Caucasians) and populations (high-risk groups with hypertension, diabetes, heart disease, and healthy low-risk groups).

#### 2.8.4 Sensitivity analysis

In order to test the stability of the meta-analysis results, we will use the one-by-one exclusion method to analyze the sensitivity of the results through Stata 16.0.

#### 2.8.5 Assessment of reporting biases

We will use a funnel chart to qualitatively identify publication bias, and use Egger and Begg tests to quantitatively evaluate publication bias. If the funnel diagram is asymmetrical and *P* *<* . 05, it is considered to have obvious publication bias.

#### 2.8.6 Evidence quality evaluation

For research that can realize meta-analysis, we will use Grading of Recommendation Assessment, Development, and Evaluation (GRADE) scoring method to grade the evidence of the outcome index.^[14]^ The evaluation content includes bias risk, indirectness, inconsistency, inaccuracy and publication bias, and the quality of evidence will be rated as high, medium, low, or very low.

## 3 Discussion

Atherosclerosis can increase cardiac afterload, reduce coronary artery perfusion, cause mechanical fatigue of the arterial wall, lead to leaving ventricular hypertrophy, myocardial ischemia and heart failure, and promote the formation of atherosclerosis and ventricular aneurysm.^[15]^ Studies have shown that the degree of arteriosclerosis is directly related to the severity of left ventricular insufficiency and can be used as an independent predictor of left ventricular diastolic function.^[16,17]^ As an independent risk factor for cardiovascular events, the risk assessment of cardiovascular events is of great significance for early diagnosis, treatment, and primary prevention of cardiovascular events.

Arteriosclerosis has a moderate genetic correlation. So far, many genes have been found to be related to the occurrence and development of arteriosclerosis.^[3]^ There are many studies on the relationship between genes encoding various components of renin-angiotensin system and arteriosclerosis, but the results are lack of consistency due to differences in sample size and research methods. Angiotensinogen produces angiotensin I under the action of renin, and then produces active angiotensin II catalyzed by angiotensin converting enzyme. By binding to AT1R, angiotensin II regulates cell proliferation, extracellular matrix deposition, and inflammation, thus affecting the structure and function of large arteries. Although the AGTR1 A1166C polymorphism is located in the non-coding region of the AT1R gene, it is related to the density of AT1R.^[18]^ In recent years, there are more and more studies on the effect of A1166C polymorphism on arteriosclerosis, but the results are inconsistent. The reason for the inconsistency is that the research scheme itself is still affected by population characteristics or other factors. This study will explore the relationship between angiotensin II 1 receptor gene polymorphism and arteriosclerosis by means of systematic review and meta-analysis. PWV can reflect the elastic function of the artery as a whole, and it is widely accepted and used as the gold standard for evaluation of arteriosclerosis.^[19,20]^ Therefore, in this study, we use PWV as the evaluation index of arteriosclerosis.

Due to the limitation of language retrieval, we will only include Chinese and English literature in this study, and other languages may be ignored. Different race, skin color, and disease and other factors may cause a certain degree of clinical heterogeneity.

## Author contributions

**Data curation:** Zhongping Shi, Jun Wang.

**Funding acquisition:** Yiwei Huang.

**Resources:** Jun Wang, Shanjiang Chen.

**Software:** Shanjiang Chen, Haiyue Dai.

**Supervision:** Shanjiang Chen.

**Writing – original draft:** Zhongping Shi, Jun Wang.

**Writing – review & editing:** Zhongping Shi, Yiwei Huang.

## Abbreviations

Abbreviations: AT1R = angiotensin II type 1 receptor, PWV = pulse wave velocity.
